# Editorial: *90th anniversary of the 1932 Sherrington* and *Adrian Nobel prize*: molecular pathways of synaptic transmission regulation

**DOI:** 10.3389/fnmol.2023.1271369

**Published:** 2023-08-22

**Authors:** Miriam Kessi, Jing Peng, Fang He, Fei Yin, Samira G. Ferreira, Xiaofei Wei

**Affiliations:** ^1^Xiangya Hospital, Central South University, Changsha, China; ^2^Clinical Research Center for Children Neurodevelopmental Disabilities of Hunan Province, Changsha, China; ^3^Centre for Neuroscience and Cell Biology, University of Coimbra, Coimbra, Portugal; ^4^David Geffen School of Medicine, University of California, Los Angeles, Los Angeles, CA, United States

**Keywords:** synaptic density, synaptic transmission, molecular pathways, synaptic plasticity, neurological diseases

In human brain, there are about 86 billion neurons that connect each other to form sophisticated networks (Li and Sheng, 2022). The communication between neurons largely relies on synaptic transmission. A classic neuronal synaptic transmission process begins with the formation of action potential which depolarizes presynaptic membranes to activate the voltage-gated calcium channels, and this will result in triggering the exocytosis of the synaptic vesicles and release of the neurotransmitters into the synaptic junction (Chapman, 2018; Madrigal et al., 2019). At the postsynaptic structure, specialization senses neurotransmitters via diverse receptors including cell-adhesion molecules (Jang et al., 2017; Südhof, 2018).

Impaired interactions of the trans-synaptic cell-adhesion molecules have been implicated in neuropsychiatry disorders (Südhof, 2018). Synaptic plasticity, a vital contributor to the long-term activity-dependent changes in neural circuits, plays a significant role in processes such as learning and memory (Südhof, 2018), specifically, N-methyl-D-aspartate receptor (NMDAR) -dependent long term potentiation has shown to be largely involved (Südhof, 2018). Energy is also a crucial component of synaptic transmission (synaptoenergetics). Dysregulation of energy regulation in synaptic transmission is linked to several neurological disorders, involving bioenergetics failure and synaptic dysfunction (Li and Sheng, 2022). Synaptoenergetics failure can cause conditions such as Parkinson's disease, amyotrophic lateral sclerosis, Alzheimer's disease, Huntington's disease, Hereditary spastic paraplegia, Progressive myoclonus epilepsy, Schizophrenia, Tuberous sclerosis complex, Charcot–Marie–Tooth disease type 2A, Dominant optic atrophy, and Fmr1- KO mice (Li and Sheng, 2022). Overall, deficit in synaptic transmission can lead to diseases such as epilepsy, intellectual disability/global developmental delay, attention-deficit/hyperactivity disorder, depression, and autism spectrum disorder (ASD) (Fukata and Fukata, 2017; Telias, 2019; Kessi et al., 2020, 2021, 2022a,b; Yan and Rein, 2022).

The aim of this Research Topic is to shed light on the current advances in understanding the basic mechanisms of neuron function, action potential generation, signal integration and transmission across the entire central nervous system. This will further enhance our comprehension of how biological mechanisms and physiological properties contribute to the global information processing. The Research Topic of five articles provides valuable updates and insights into various aspects related to synaptic transmission.

In one study, a mutation (c.892C>T, p.Arg298Trp) in the NACC1 gene was linked to severe neurological symptoms including epilepsy and intellectual disability. Investigating this novel association, Daniel et al. explored how the mutation could alter brain function by examining the neurotransmission in glutamatergic mouse neurons expressing the human mutant NACC1 (Nacc1-R284W). They observed that the expression of Nacc1-R284W in mouse impaired glutamatergic neurotransmission in a cell-autonomous manner. In addition, they discovered (SYNaptic GTPase Activating Protein) SynGAP1, glutamate kainate receptor subunit 2 (GluK2), and several small ubiquitin-like modifier (SUMO) protein ligases (E3) (SUMO E3) as novel Nacc1 interactive proteins. Nacc1-R284W displayed reduced binding capacity to SynGAP1 and GluK2, and augmented SUMOylation. Their findings suggest a role for Nacc1 in regulating glutamatergic neurotransmission (Daniel et al.).

Another study by Ramsay et al. assessed the synaptic nano-architecture under situations where presynaptic neurotransmitter discharge was blocked before and during synaptogenesis. Their study emphasized that the neurotransmitter release was not mandatory for the formation of excitatory or inhibitory synapses. Despite this, basic features of synaptic nano-architecture, including assembly of receptors and scaffolds into trans-synaptically aligned structures, were found to be intrinsic properties that can be further regulated by subsequent activity-dependent mechanisms (Ramsay et al.).

Liu et al. explored the mechanism by which glucagon-like peptide-1 (GLP-1) controls mouse cerebellar Purkinje cell activity *in vitro*. Their study revealed that GLP-1 plays an important role in moderating cerebellar function by regulating the spike firing activity of mouse cerebellar Purkinje cell (Liu et al.).

Furthermore, Bykhovskaia reviewed and discussed how Drosophila transgenic lines that express postsynaptically tethered calcium ions sensor GCaMP enables the exploration of the evoked and spontaneous transmission at single active zones. This review helps the understanding of the properties of both release components, including decoupling between the evoked and spontaneous release (Bykhovskaia).

Lastly, Ge and Wang summarized the GluN2B-containing NMDAR pharmacology and its key physiological functions, emphasizing its importance during both health and disease states. They summarized the role of the GluN2B-containing NMDAR in intellectual disability, ASD, schizophrenia, stroke, traumatic brain injury, epilepsy, major depressive disorder, Alzheimer's disease, Parkinson's disease, and Huntington's disease (Ge and Wang).

Altogether, this Frontier in Molecular Neuroscience Research Topic provides updates on various aspect of glutamatergic neurotransmission, GluN2B-containing NMDAR pharmacology in health and disease states, basic features of synaptic nano-architecture, mechanism of GLP1 in cerebellar function exploration of evoked and spontaneous transmission. We express our gratitude to each author and hope that this Research Topic will encourage further exploration of the molecular mechanisms in synaptic transmission regulation.

## Author contributions

MK: Conceptualization, Project administration, Supervision, Validation, Writing—original draft, Writing—review and editing. JP: Writing—review and editing, Validation. FH: Writing—review and editing, Validation. FY: Writing—review and editing, Validation. SF: Writing—review and editing, Validation. XW: Writing—review and editing, Validation.
